# Histopathological Alterations in Gills, Liver and Kidney of African Catfish (*Clarias gariepinus*, Burchell 1822) Exposed to *Melaleuca cajuputi* Extract

**DOI:** 10.21315/tlsr2023.34.2.9

**Published:** 2023-07-21

**Authors:** Marina Hassan, Anuar Abdalah Nagi Melad, Mohd Ihwan Zakariah, Nor Asma Husna Yusoff

**Affiliations:** 1 Higher Institution Centre of Excellence (HICoE), Institute of Tropical Aquaculture and Fisheries, Universiti Malaysia Terengganu, 21300 Kuala Nerus, Terengganu, Malaysia; 2 Department of Biological Science, Faculty of Science, Azzaytuna University, Tarhunah, Libya

**Keywords:** Gelam, Catfish, Histopathology Alterations, Sub-Lethal, Gelam, Ikan Keli, Perubahan Histopathologi, *Sub-Lethal*

## Abstract

This study evaluated the histopathological changes in the gill, liver and kidney of African catfish (*Clarias gariepinus*) intoxicated with a sub-lethal dose of *Melaleuca cajuputi* leaves extract (MCLE) for 96 h. The acute toxicity test has been determined previously with a value of 96-h LC_50_ = 127 mg/L, hence the selection of sub-lethal ranges from 60 mg/L to 160 mg/L of MCLE. Degenerative alterations were prominent in all tested organs, particularly after exposure to a high concentration of MCLE. Gill exhibited haemorrhage, epithelial lifting, lamellar disorganisation, and necrosis after exposure to a high MCLE concentration. Alterations in the liver include congestion, hydropic degeneration, and vacuolation, whereas lesions in the kidney were pyknosis, vacuolation, hydropic degeneration, and tubular necrosis. The obtained data showed that the organs experienced severe changes proportional to the increase in MCLE concentration. In addition, fish exposed to higher concentrations than the LC_50_ value experienced irreversible lesions. The present study suggests that the use of MCLE below the LC_50_ is recommended to avoid severe alterations to organs, particularly in African catfish. This study demonstrated that the use of MCLE above the LC_50_ promotes severe damage to the gills, liver and kidney of African catfish. However, further investigations are needed to define the causing-mechanisms underlying these effects.

## Abstrak

Kajian ini menilai perubahan histopatologi dalam insang, hati dan buah pinggang ikan keli Afrika (*Clarias gariepinus*) yang ditoksikkan dengan dos *sub-lethal* ekstrak daun *Melaleuca cajuputi* (MCLE) selama 96 jam. Ujian ketoksikan akut telah ditentukan sebelum ini dengan nilai 96-jam LC_50_ = 127 mg/L, oleh itu pemilihan julat *sub-lethal* dari 60 mg/L hingga 160 mg/L MCLE. Perubahan degeneratif adalah ketara dalam semua organ yang diuji, terutamanya selepas terdedah kepada kepekatan MCLE yang tinggi. Insang menunjukkan pendarahan, pengangkatan epitelium, kecelaruan lamellar, dan nekrosis selepas terdedah kepada kepekatan MCLE yang tinggi. Perubahan dalam hati termasuk kesesakan, degenerasi hidropik dan vakuolasi, manakala luka pada buah pinggang adalah piknosis, vakuolasi, degenerasi hidropik dan nekrosis tiub. Data yang diperolehi menunjukkan bahawa organ-organ mengalami perubahan teruk yang berkadar dengan peningkatan kepekatan MCLE. Selain itu, ikan yang terdedah kepada kepekatan yang lebih tinggi daripada nilai LC_50_ mengalami luka yang tidak dapat dipulihkan. Kajian ini mencadangkan bahawa penggunaan MCLE di bawah nilai LC_50_ disyorkan untuk mengelakkan perubahan teruk pada organ, terutamanya pada ikan keli Afrika. Kajian ini menunjukkan bahawa penggunaan MCLE di atas nilai LC_50_ menggalakkan kerosakan teruk pada insang, hati dan buah pinggang ikan keli Afrika. Walau bagaimanapun, penyiasatan lanjut diperlukan untuk menentukan mekanisma punca yang menjadi penyebab kepada kesan ini.

**Highlights**

- The severity of gills, liver and kidney of African catfish were proportional to the increase of *Melaleuca cajuputi* leaves extract (MCLE) concentrations.
- Degeneration alterations were notable in gills, liver and kidney of African catfish when exposed to MCLE concentration over than the LC_50_ value.
- It was recommended that MCLE exposure be less than the LC_50_ value to lessen the histopathological alterations in the gills, liver and kidneys of African catfish.

## INTRODUCTION

Aquaculture is one of the fastest growing sectors in the food industry, accounting for more than 20% of all animal protein exported to low-income countries worldwide (Tacon 2020). Among them, fish farming accounts for nearly 40% (54.3 million tonnes), followed by mollusks and crustaceans at 17.7 million tonnes and 9.4 million tonnes, respectively (FAO 2020). However, intensive aquaculture with high stocking capacity promotes the spread of pathogenic microorganisms, resulting in massive economic losses (Valenzuela-Gutiérrez *et al*. 2021). Previously, several chemical antibiotics such as formalin, trichlorfon and mebendazole were used to control this situation (Liu *et al*. 2021), but their overuse resulted in antibiotic accumulation, which might pose health risks to the consumer (Aisiah *et al*. 2020). Furthermore, long-term use of these antibiotics may result in bacterial disease resistance and a lack of efficacy in some control agents (Zhang *et al*. 2014). To avoid the use of chemotherapeutical treatments, fish need to improve their immunity to combat the dispersion of diseases and pathogens. The use of plant extracts as immunostimulants in aquatic organisms has been reported worldwide through improving growth and body defense mechanisms (Sahimi *et al*. 2022; Doan *et al*. 2022; Rashidian *et al*. 2021; Nafiqoh *et al*. 2020; Mohammadi *et al*. 2020). Therefore, the use of plant extracts as an alternative is promising as a potential strategy to improve fish immunity to combat diseases and parasites due to their advantages of being environmentally friendly and biodegradable (Liu *et al*. 2021).

*Melaleuca cajuputi* is one of the native plants that are commonly found throughout Southeast Asia and Australasia (Nagi *et al*. 2016). Major phytochemical compounds such as terpenes, alkaloids, phenolics and flavonoids were previously reported in *M. cajuputi* extract which contribute to a broad-spectrum of biological activities (Chaudhari *et al*. 2022; Sahimi *et al*. 2022; Septiana *et al*. 2020; Bua *et al*. 2018; Chan & Haron 2016; Al-Abd *et al*. 2015). Some of them demonstrated their toxicity through acute toxicity tests (Jayakumar *et al*. 2020; Noor *et al*. 2021; Bakar 2020; Roszaini *et al*. 2013), but not all of the activity has been effectively characterised in vivo to determine their safety effect at a sub-lethal dose. There are few reports of histopathological changes following the use of plant extracts on aquatic organisms (Meneses *et al*. 2020; Karataş *et al*. 2020; Meneses *et al*. 2018; Shivappa *et al*. 2015), but there is a paucity in the *M. cajuputi* plant extract. Therefore, this study was performed to determine the histopathological changes occur in the gills, liver, and kidney of African catfish at sub-lethal doses. Earlier, an acute toxicity study of *Melaleuca cajuputi* leaves extract (MCLE) had been done on the catfish, *Clarias gariepinus*, which gave a 96-hour LC_50_ of 127 mg/L (Nagi *et al*. 2016). The findings may be useful in determining the safety of using MCLE as a baseline for fish, particularly African catfish, as therapeutics.

## MATERIALS AND METHODS

### Collection and Identification of *M. cajuputi* Powell

Fresh *Melaleuca cajuputi* leaves were collected in Marang, Terengganu (5.4932°N, 102.9320°E) and sent to botanist for taxonomy identification under the voucher number of MFI 0244/22.

### Preparation of *M. cajuputi* leaves Extract (MCLE) and Treatment Solution

Leaves were washed under running tap water to remove any soil debris and dried at room temperature (27 ± 2°C) for 14 days. Dried leaves were ground and weighed for 10 g before soaking in 80% methanol for 24 h with shaking (27°C, 200 rpm). The mixture was then filtered using filter paper (Whatman, UK) and the filtrate was further concentrated using a rotary evaporator (Buchi Rotavapor-R, Fisherscience, UK) at 38°C until the gummy-like crude extract was formed. The crude extract was freeze-dried and the powder form was stored at −20°C until further use.

### Phytochemical Compound Identification Using Gas Chromatography-Mass Spectra (GC-MS) Analysis

GC-MS analysis was performed according to Al-Abd *et al*. (2015), with slight modifications. Briefly, 10 mg of MCLE sample was sonicated for 15 min in 2.5 mL of dichloromethane at 40°C in a sealed vial. Then, 1 mL of the mixture was filtered through a 0.20 μM nylon filter, and 1 μL of the filtrate was injected splitless into the GC-MS system (Hawlett-Packard autosampler 6890 GC, Agilent, USA), equipped with a capillary column (diameter: 30 m × 0.25 mm; thickness: 0.24 μM). The column initial temperature was initially set up at 40°C for 3 min, and the final temperature was at 280°C for 3 min. Helium was used as a carrier gas at 1 mL/min flow rate. The ion source temperature and injector were both maintained at 250°C. The mass spectrometer was scanned over the range of 28–400 m/z amu at 1 scan^−1^, with an ionising voltage of 70 eV. The spectra that came up were then compared with the known compounds from the NIST database library based on their retention time (RT) index of primary and secondary metabolites.

### Fish Acclimatisation

*Clarias gariepinus* with a mean body weight and total length of 20.0 ± 3.0 g and 15.0 ± 2.0 cm, respectively, were obtained from the hatchery at the Institute of Tropical Aquaculture and Fisheries, Universiti Malaysia Terengganu (UMT). Fish were placed in a tank containing 500 L of non-chlorinated water and acclimatised for two weeks under laboratory conditions. The fish were fed daily with commercial fish pellets at 3% of their body weight. The water quality parameters, including temperature, pH and dissolved oxygen, were measured using multiparameter equipment (YSI 556 MPS, USA) and maintained at 26°C–28°C, 5.50–6.25 and >5 mg/L, respectively. Approximately 80%–90% of the water was changed daily to keep the quality.

### Experimental Procedure

The acute toxicity and behavioral studies have been performed previously by Nagi *et al*. (2016), which indicated the 96-hour LC_50_ value of MCLE to be at 127 mg/L. In this present study, a sub-lethal toxicity test was performed to determine the histological changes on gills, liver and kidney of African catfish after exposure to MCLE for 96 h, as according to Correia *et al*. (2020), with slight modifications. Briefly, six MCLE concentration were used in this study, (i.e., 0.0, 60, 80, 100, 120, 140 and 160 mg/L), where 0.0 mg/L was served as control. Healthy acclimatised fish were randomly distributed in 18 aquaria (50 L) containing MCLE solutions, with 5 fish per treatment, and in triplicates (5 fish × 6 MCLE concentrations × 3 replicates). Water parameters including temperature, pH and dissolved oxygen were controlled at 26°C–28°C, 5.5–6.25 and >5 mg/L, respectively using YSI multi-parameter probe (YSI Inc., USA). No feeding was performed during the experiment.

### Histological Assessment of Gills, Liver and Kidney of African Catfish

At the end of experiment, fish were euthanised by rapid cooling (Rodrigues *et al*. 2019), decapitated, and the gills, liver and kidney were carefully removed. Thereafter, the organ tissues were fixed in 10% formalin solution, followed by dehydration through six grades of alcohol separately, sectioned using a microtome (Leica, Germany) sectioning, and stained with H&E. Finally, the mounted slides were examined under a light microscope (Leica, Germany) to observe any histological changes in the tissues. Histopathological changes observed in tissue were captured using a digital camera attached with software (Leica Application Suite V4.0, Leica, Germany) at various magnifications. The histopathological alterations were evaluated using quantitative histological assessment as adopted by Bernet *et al*. (1999), and Nero *et al*. (2006), with some modifications. Each organ were classified into four reaction patterns (rp): circulatory, degeneration, inflammatory and structural. The score values from 1 to 6 (a) were assigned based on the percentage of each alteration, whereas the importance factors (w) ranging from 1 to 3 indicate how the alterations might affect the fish health. The calculation for each category of alterations and the total pathological index were defined as:


$$\begin{array}{l} {\text{Org}}_{\text{cat}}=\Sigma_{\text{alt}}\left(a{\text{Org}}_{\text{cat}-\text{alt}}\times w{\text{Org}}_{\text{cat}-\text{alt}}\right)\\ {\text{T}}_{\text{org}}=\Sigma_{\text{cat}}\Sigma_{\text{alt}}\left(a{\text{Org}}_{\text{cat}-\text{alt}}\times w{\text{Org}}_{\text{cat}-\text{alt}}\right) \end{array}$$

where org = organ, cat = category, alt = alteration, a = score and w = importance factor.

### Statistical Analysis

Data normalities were checked prior to statistical analysis using normal probability plots. The statistical differences between the control and MCLE-exposed fish were determined using one-way analysis of variance (ANOVA), and Tukey pairwise test at *p* < 0.05 to indicate the significant difference relative to the control group (Genstat Software ver. 12.1).

## RESULTS

### Determination of Phytochemical Compounds in MCLE using GC-MS Analysis

**Figure 1:**
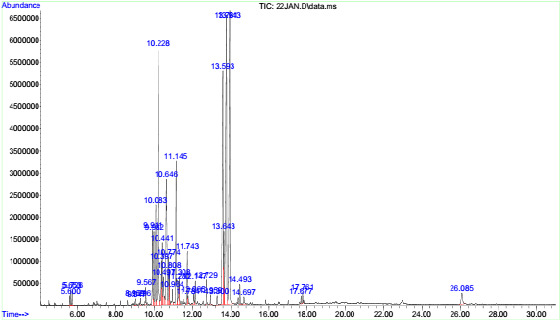
Total ion chromatogram of MCLE by GC-MS analysis.

A total of 37 compounds were identified in MCLE as shown in Table 1. There are four major compounds were detected including 1H-imidazole, 4,5-diphenyl- (18.60%), followed by 7-methyl-6,8-bis(methylthio)pyrrolo[1,2]pyrazine (15.43%), 2-isopropyl-10-methylphenanthrene (13.50%) and 9-carboxaldehyde-10-methylanthracene (11.13%) within 30 min of retention time (Fig. 1).

### Histopathological Assessment

**Figure 2:**
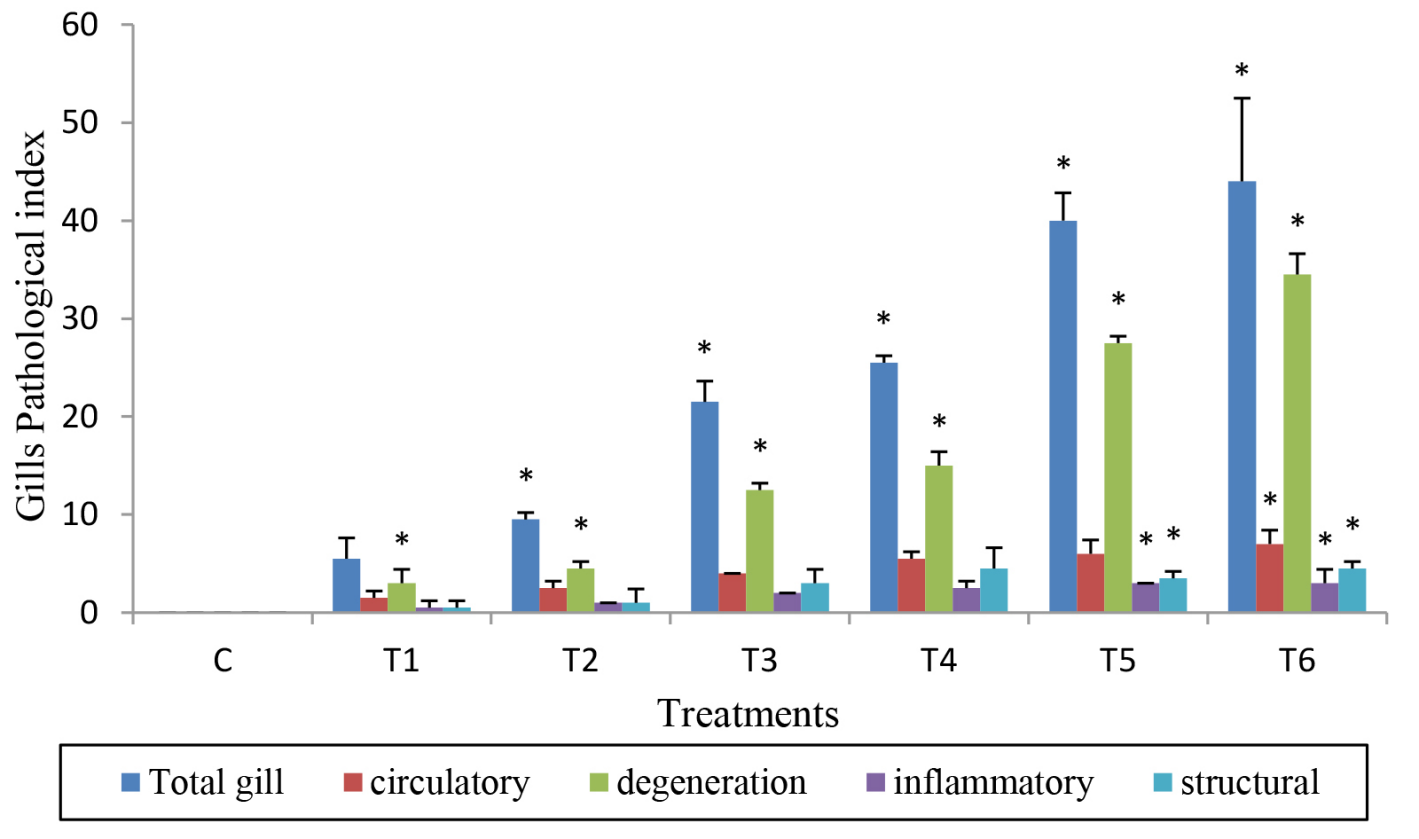

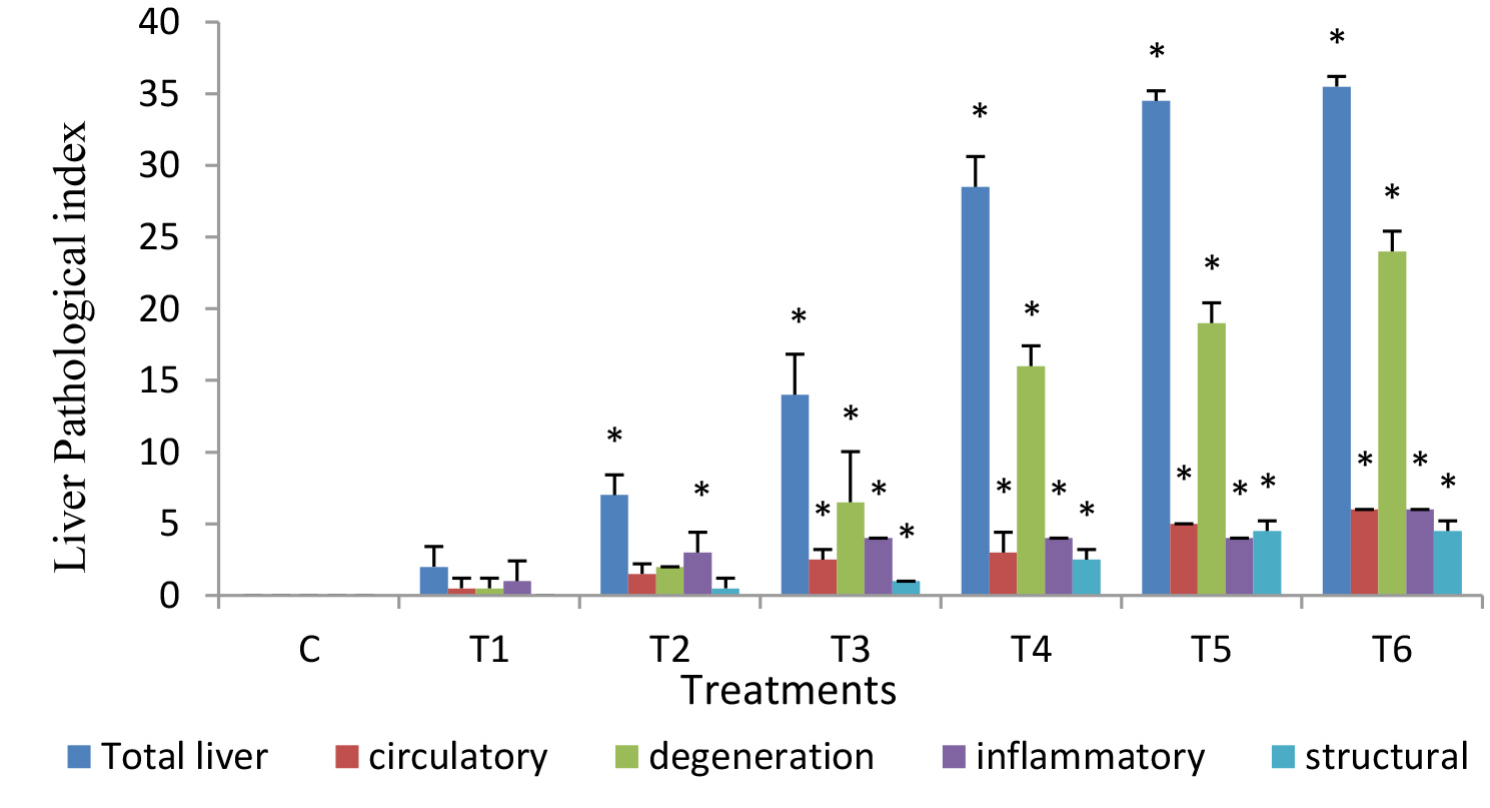

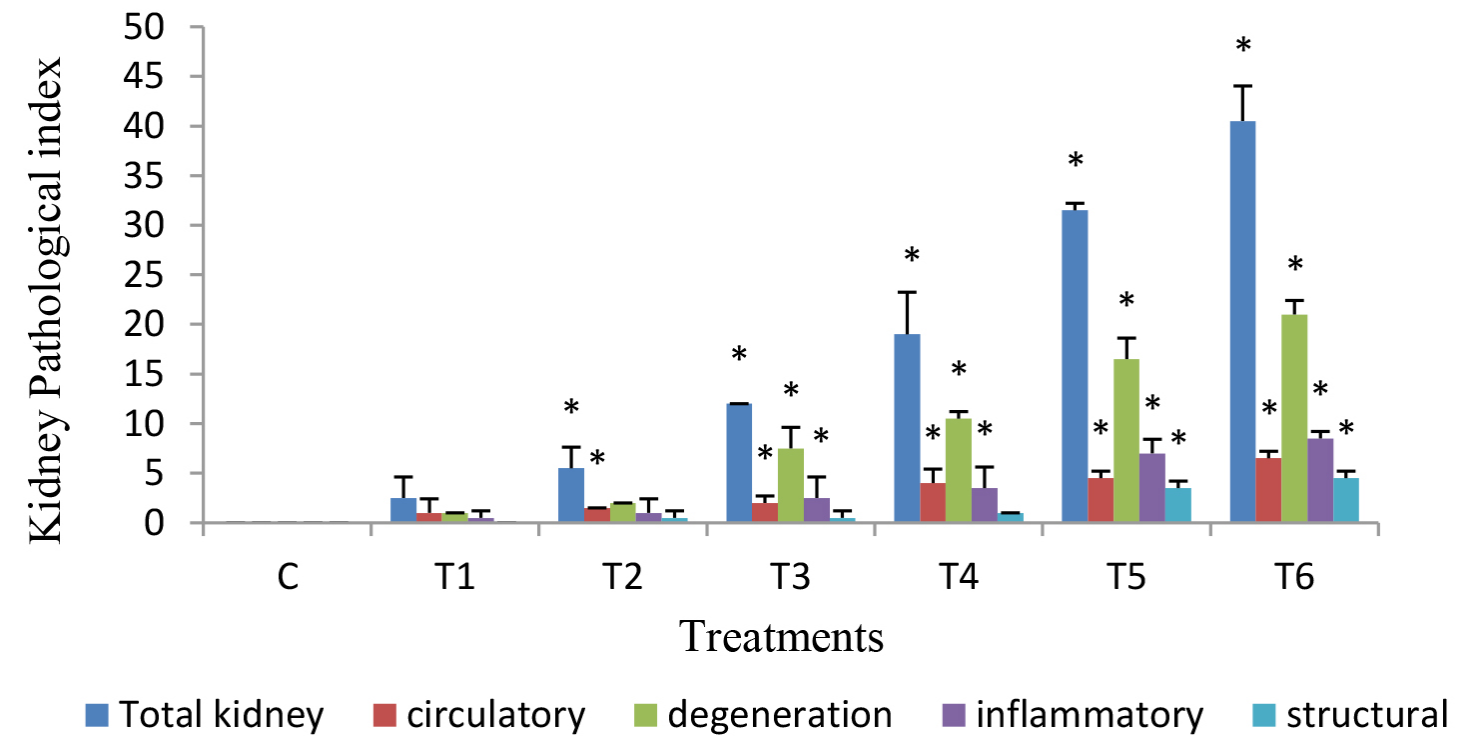
Pathological indices of C. gariepinus (A) gills, (B) liver and (C) kidney at different concentration of MCLE. C = Control; T1 = 60 mg/L; T2 = 80 mg/L; T3 = 100 mg/L; T4 = 120 mg/L; T5 = 140 mg/L and T6 = 160 mg/L. Asterisk (*) have significant different at *p* < 0.05.

Overall, the gills, liver and kidney of African catfish showed significant pathological alterations when exposed to a higher concentration of MCLE (*p* < 0.05) than the control. All organs showed pathological indices associated with the elevation of MCLE concentrations, particularly starting at 120 mg/L to 160 mg/L. Degenerative alterations predominated (*p* < 0.05) in all the tested organs of MCLE-treated fish compared to control (Figs. 2A, 2B and 2C). Concerning to gills and liver, necrosis was recorded at higher MCLE concentrations (120 mg/L to 160 mg/L), whereas started later at 140 mg/L to 160 mg/L in the kidney. However, nuclear alterations (e.g., pyknosis) and hydropic degeneration were already observed in the kidney of fish exposed to 120 mg/L. In terms of circulatory changes, sinusoids congested with red blood cells and cytoplasmatic vacuolation were observed in the liver at lower exposures of MCLE. Among the reported circulatory changes, haemorrhage was the most detected in fish kidney, even increasing the lowest exposure of MCLE concentration.

**Figure 3:**
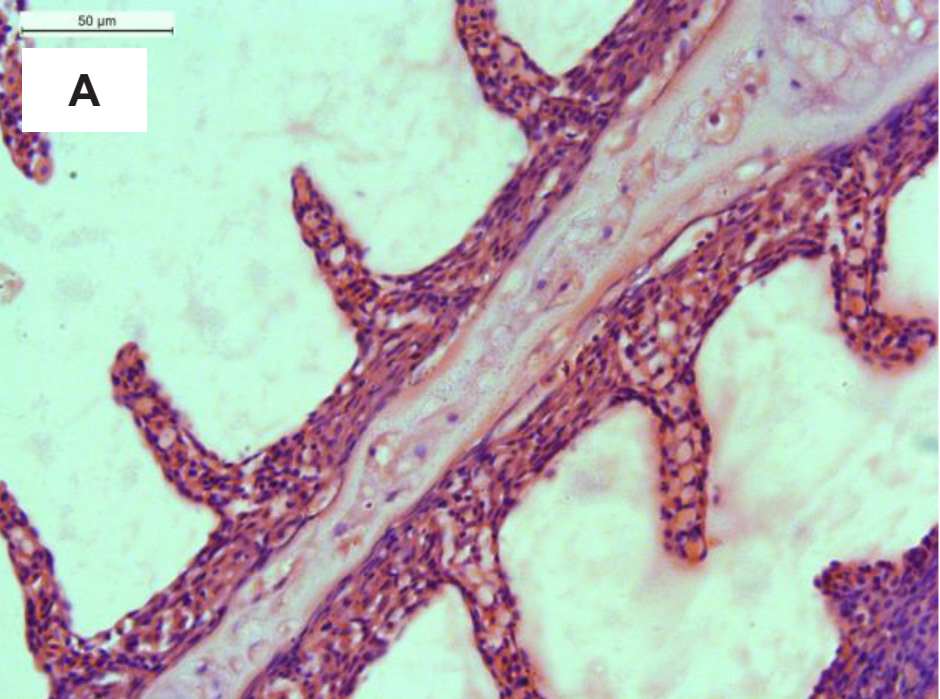

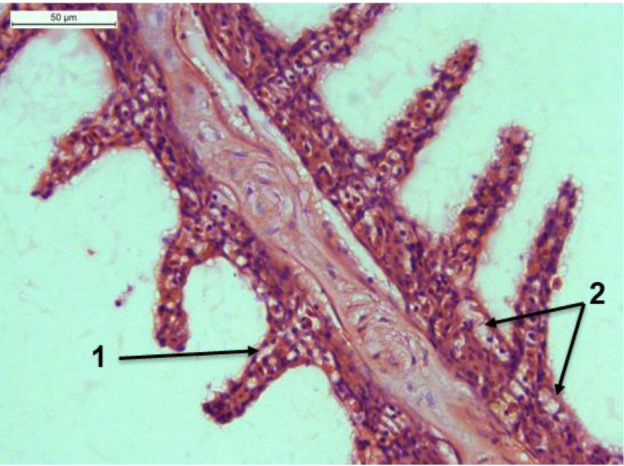

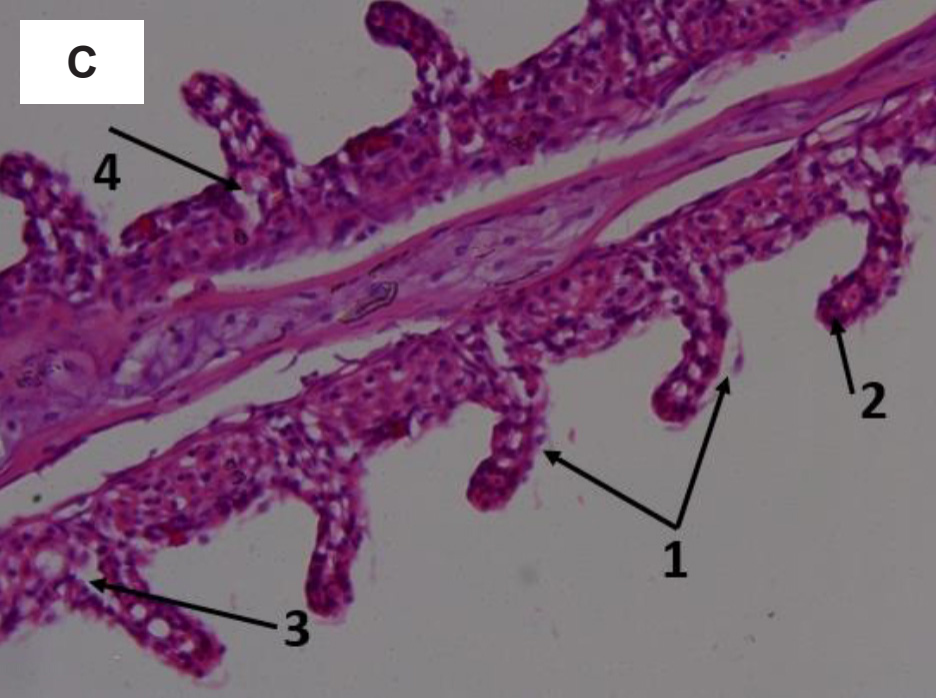

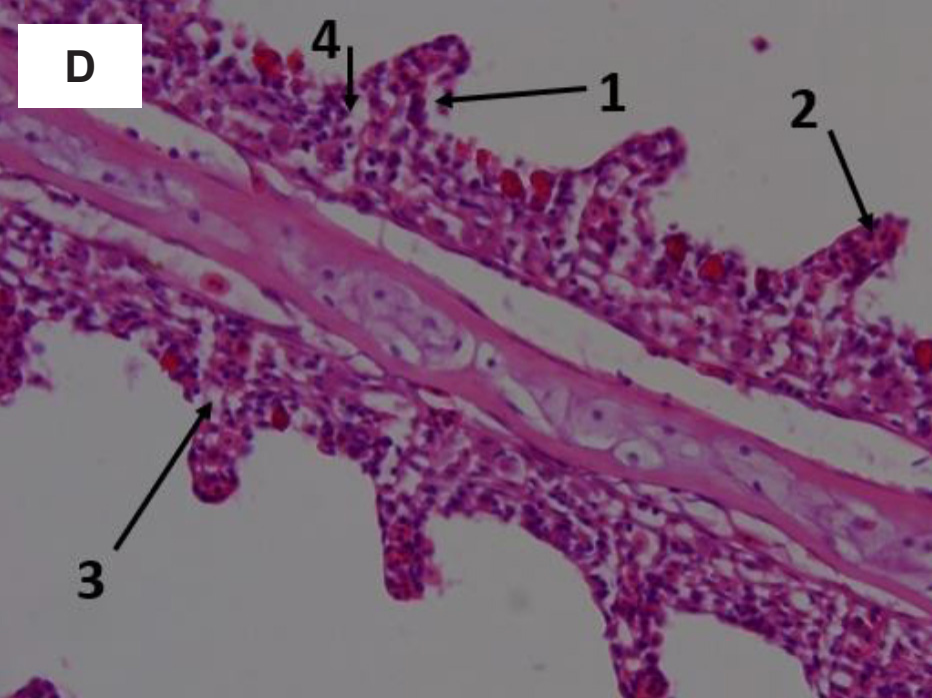

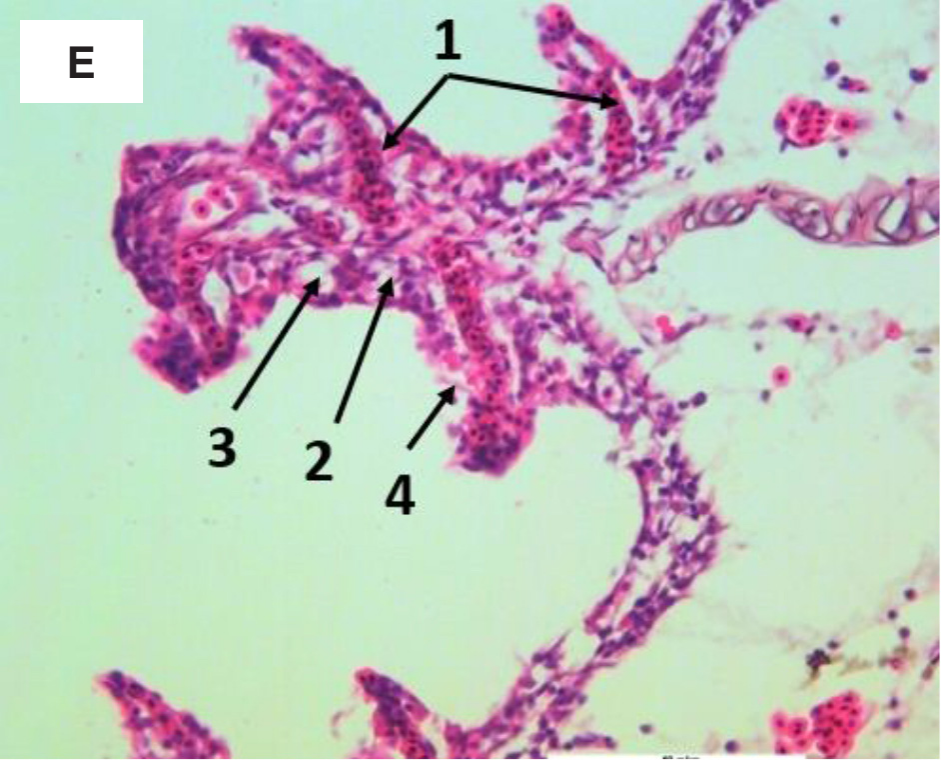

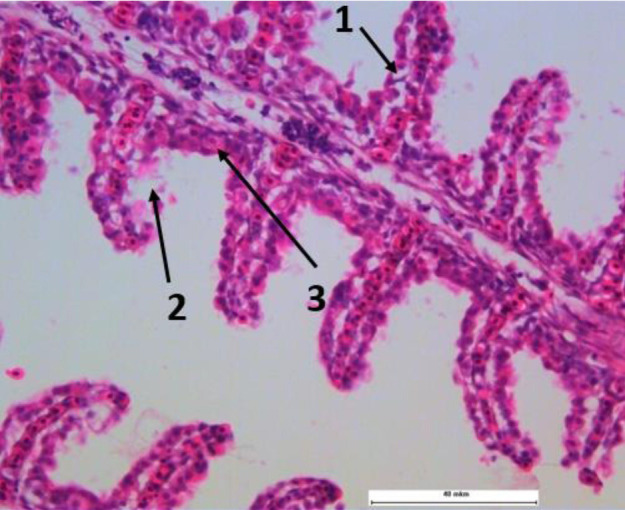

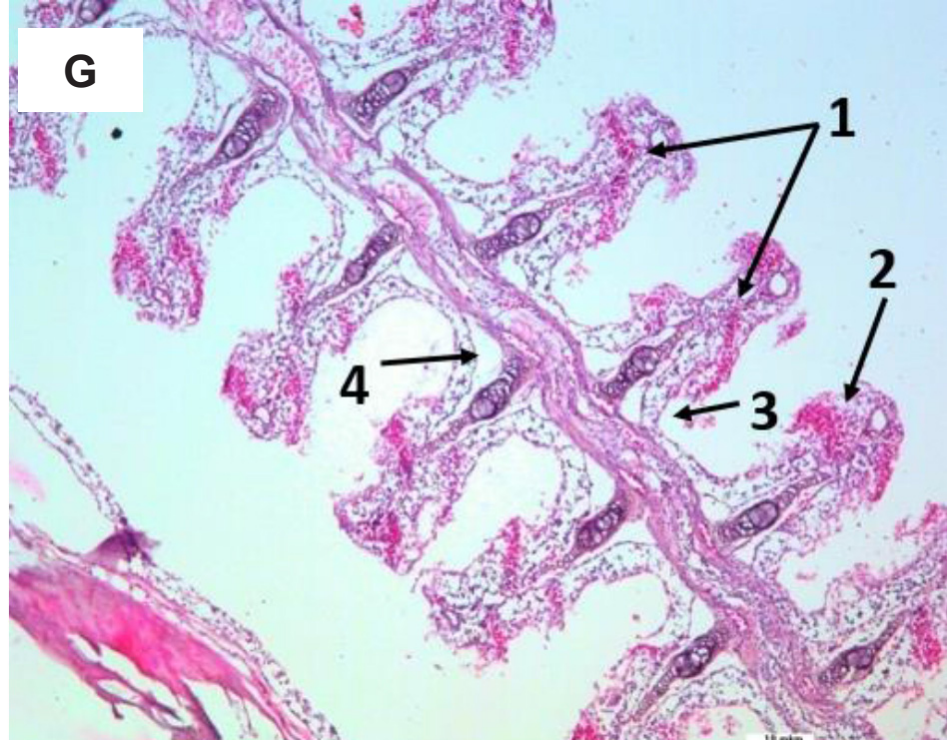
(A) Normal gills of *C. gariepinus* (0 mg/L MCLE); (B) 60 mg/L of MCLE with 1. Oedema, 2. Hydropic degeneration of the epithelial cells; (C) 80 mg/L MCLE with 1. Epithelial lifting, 2. Vines congestion, 3. Oedema, 4. Hydropic degeneration of the epithelial cells; (D) 100 mg/L MCLE with 1. Epithelial lifting, 2. Vines congestion, 3. Epithelial cells degeneration, 4. Oedema; (E) 120 mg/L MCLE with 1. Haemorrhage, 2. Lamellar epithelial cells degeneration, 3. Oedema, 4. Necrosis; (F) 140 mg/L MCLE with 1. Epithelial lifting of secondary lamellae, 2. Necrosis, 3. Hydropic degeneration of the epithelial cells; (G) 160 mg/L MCLE with 1. Lamellar disorganisation, 2. Haemorrhage, 3. Necrosis, 4. Oedema. H&E stain, 400× total magnifications.

No histological changes were observed in the gills, liver and kidney of non-treated fish (Fig. 3A). However, several changes were observed in MCLE-treated fish with the severity increasing as the plant extract concentration increased. In the 60 mg/L MCLE (Fig. 3B), a minor degree of oedema, hydropic degeneration of the epithelial cells, and lamella fusion were observed. High tissue alteration was observed in 80 mg/L MCLE (Fig. 3C), whereas lesion was observed in 100 mg/L MCLE, including epithelial lifting, congestion, lamellar epithelial cell degeneration, and oedema (Fig. 3D). Severe lesions including haemorrhage, lamella fusion, oedema, and necrosis were observed in 120 mg/L MCLE (Fig. 3E). Severe degenerative and necrotic changes in gill epithelia were detected in gills exposed to 140 mg/L and 160 mg/L MCLE (Figs. 3F, 3G), with huge lamellar disorganisation and aneurisms found only in 160 mg/L MCLE (Fig. 3G).

**Figure 4:**
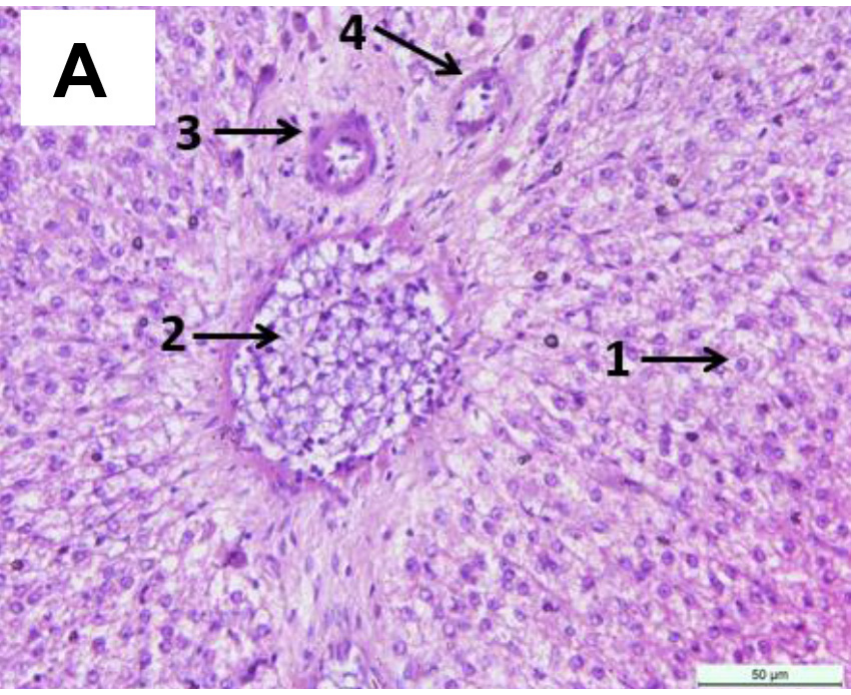

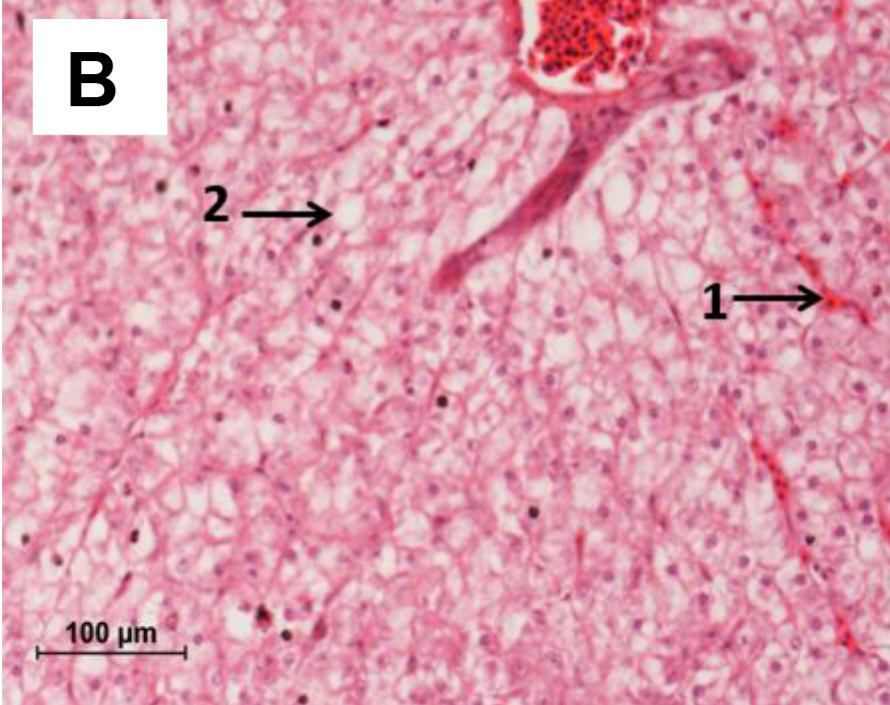

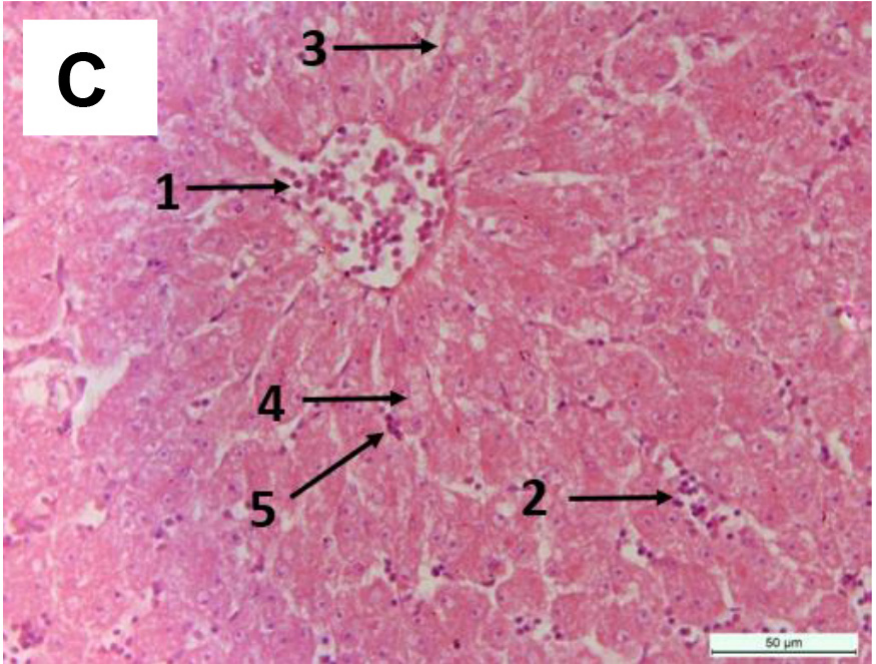

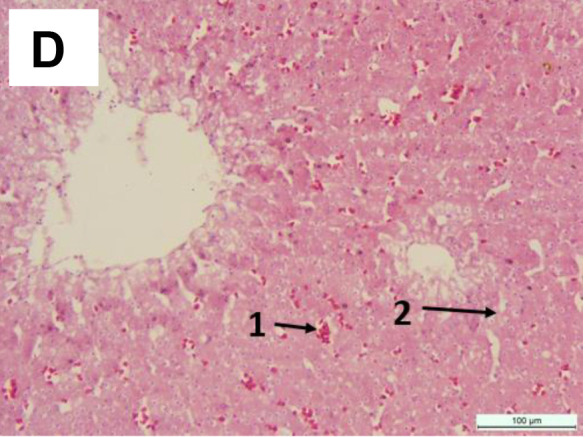

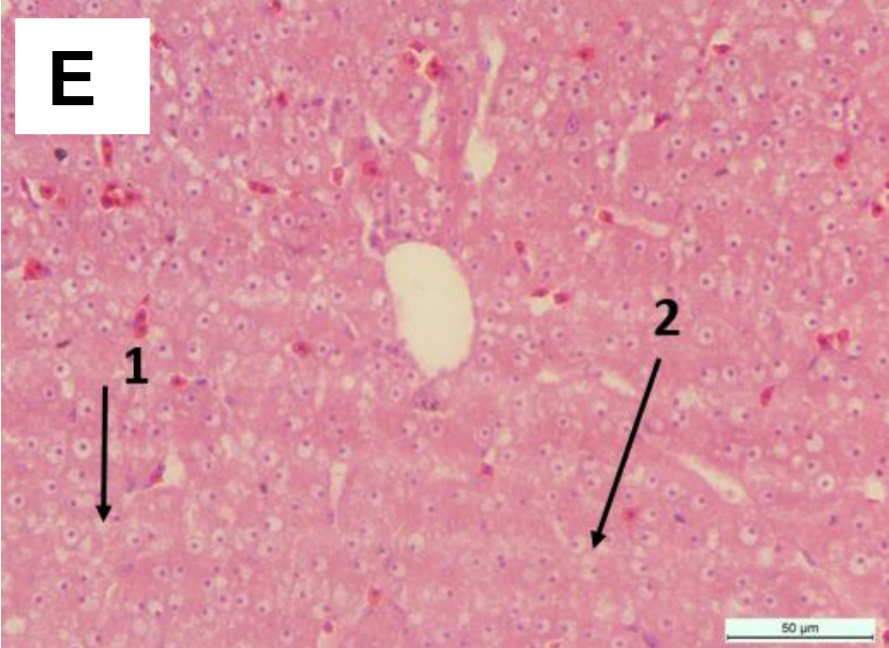

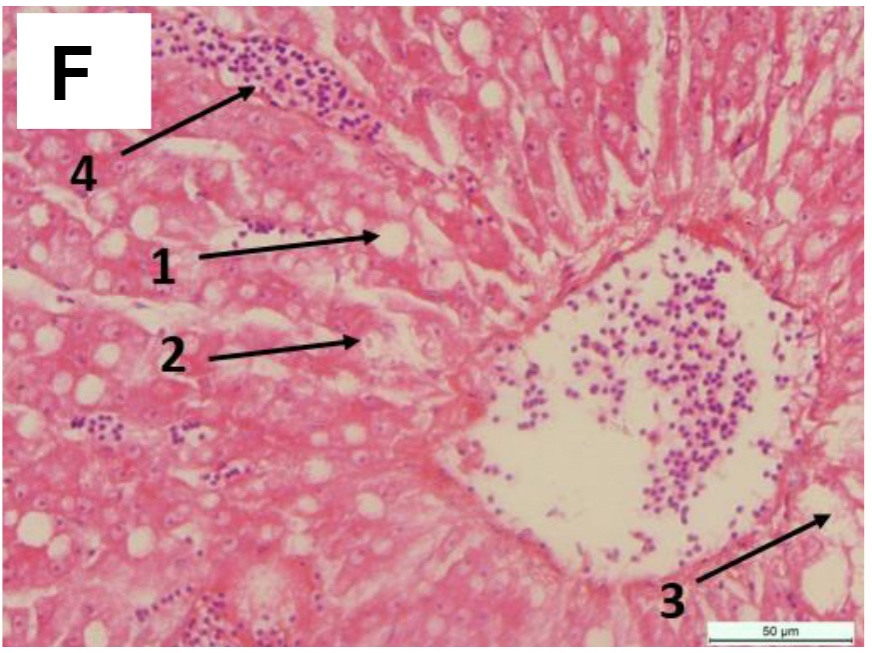

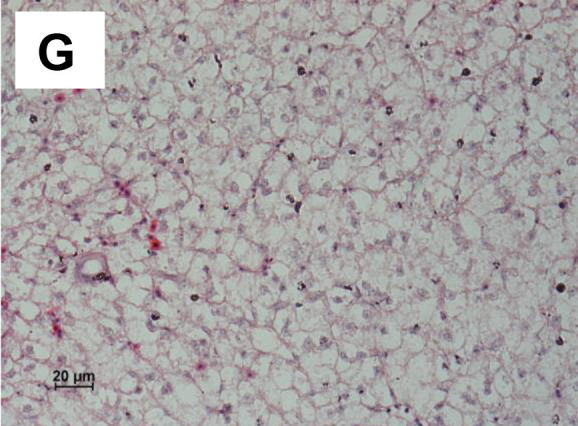
(A) Normal liver of *C. gariepinus* (0 mg/L MCLE) with 1. Hepatocytes, 2. Hepatic portal vein, 3. Hepatic artery, 4. Bile duct; (B) 60 mg/L MCLE with 1. Sinusoid congested with red blood cells, 2. Cytoplasmatic vacuolation; (C) 80 mg/L MCLE with 1. Central vein congested with red blood cells, 2. Infiltration of leukocytes, 3. Vacuolation, 4. Cloudy degeneration, 5. Aggregation of inflammatory cell between hepatocytes; (D) 100 mg/L MCLE with 1. Structure alteration; (E) 120 mg/L MCLE with 1. Focal necrosis of hepatocytes, 2. Hydropic degeneration; (F) 140 mg/L MCLE with 1. Vacuolation, 2. Degeneration, 3. Necrosis, 4. Increase in sinusoidal spaces and congested with red blood cells; (G) 160 mg/L MCLE with severely necrotic liver tissues, vacuolation of hepatocytes, hydropic degeneration of hepatocytes and poor hepatic cord structural. H&E stain, 400× total magnifications.

**Figure 5:**
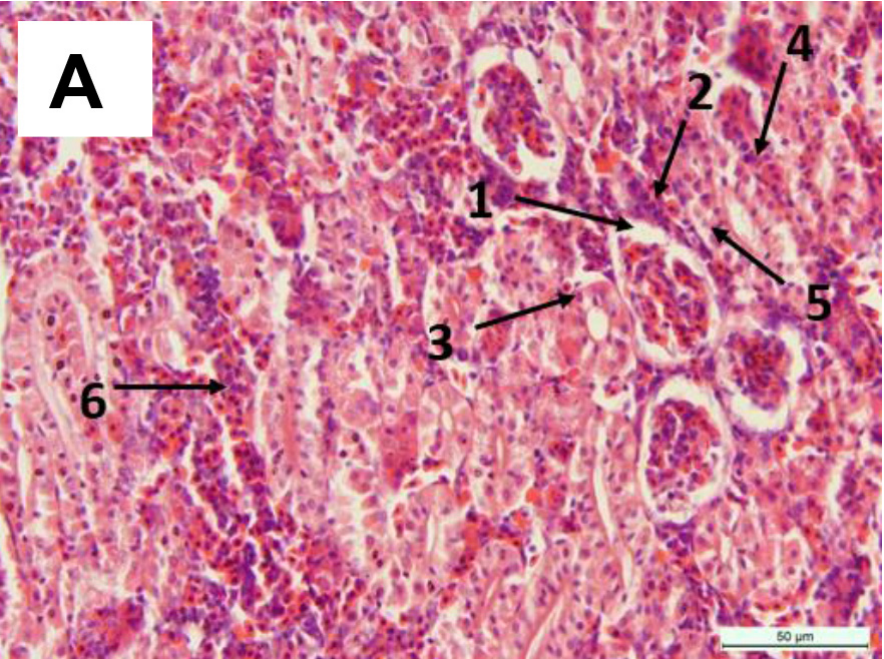

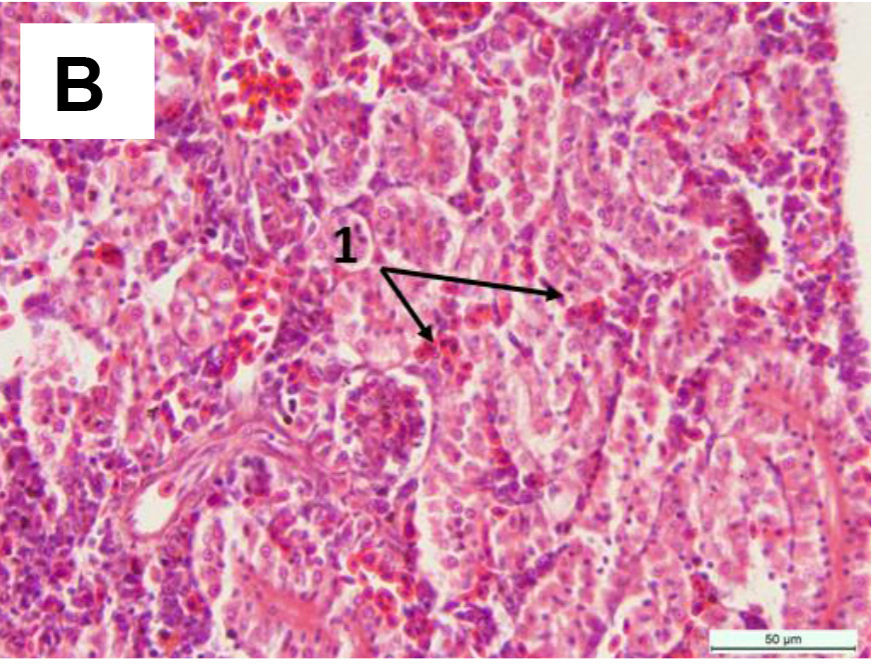

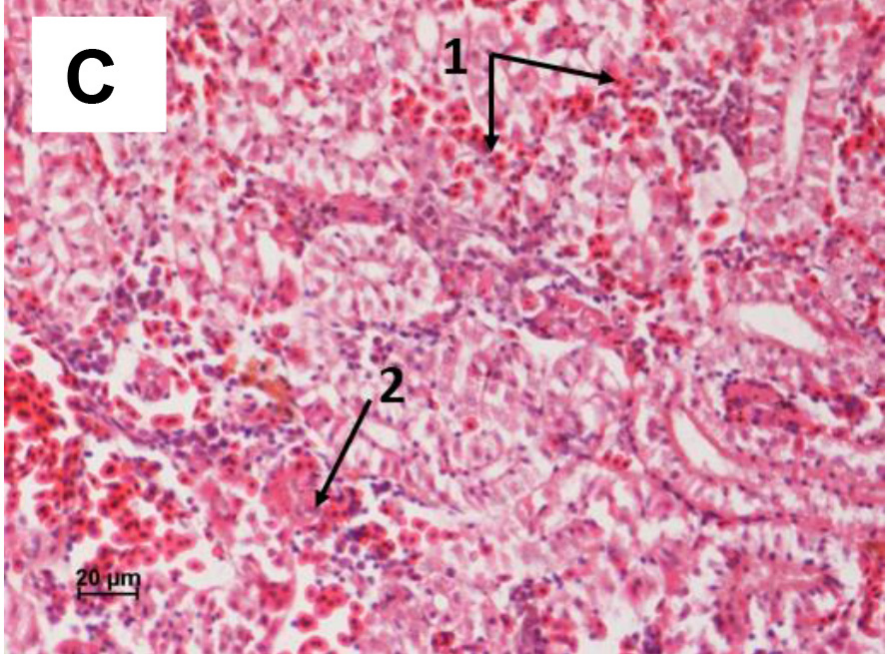

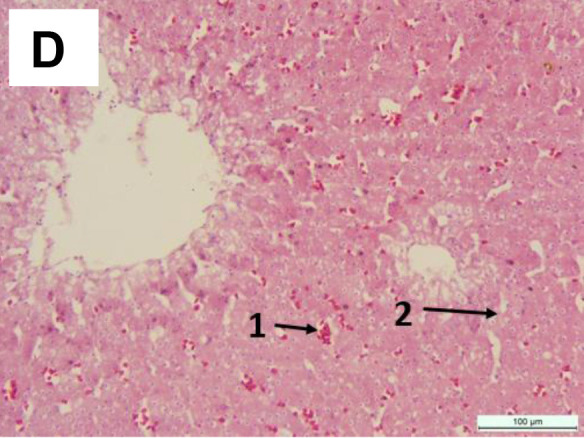

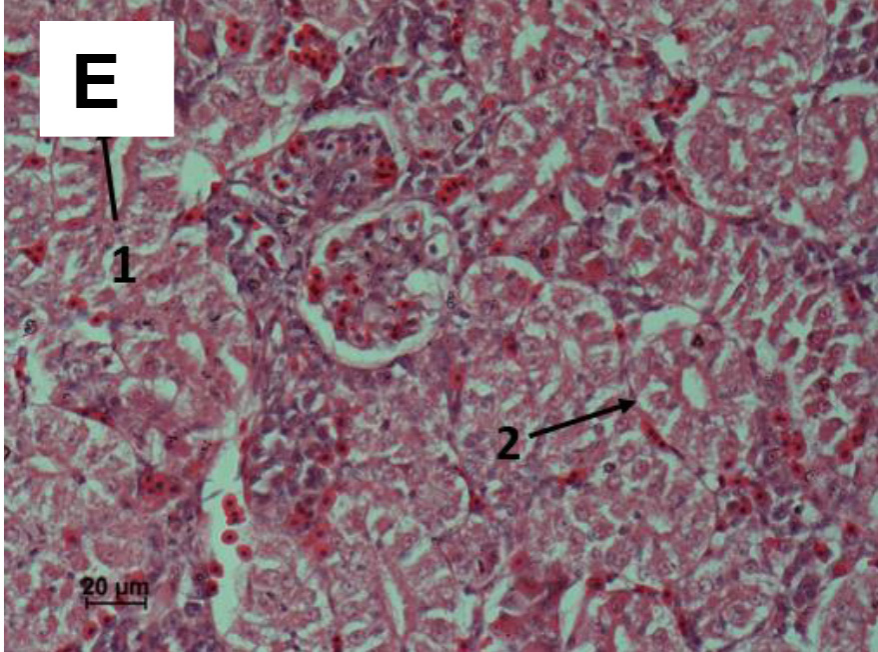

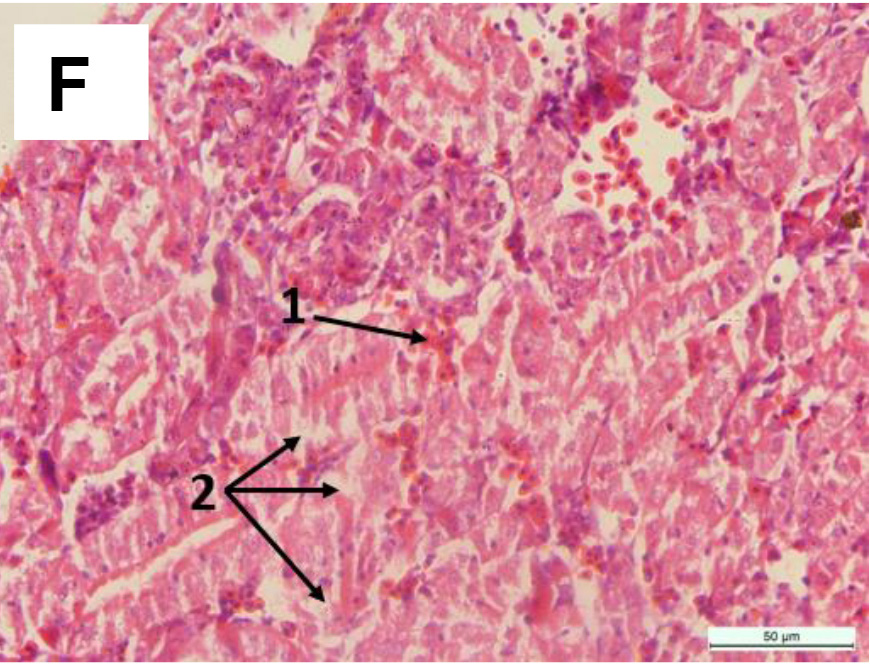

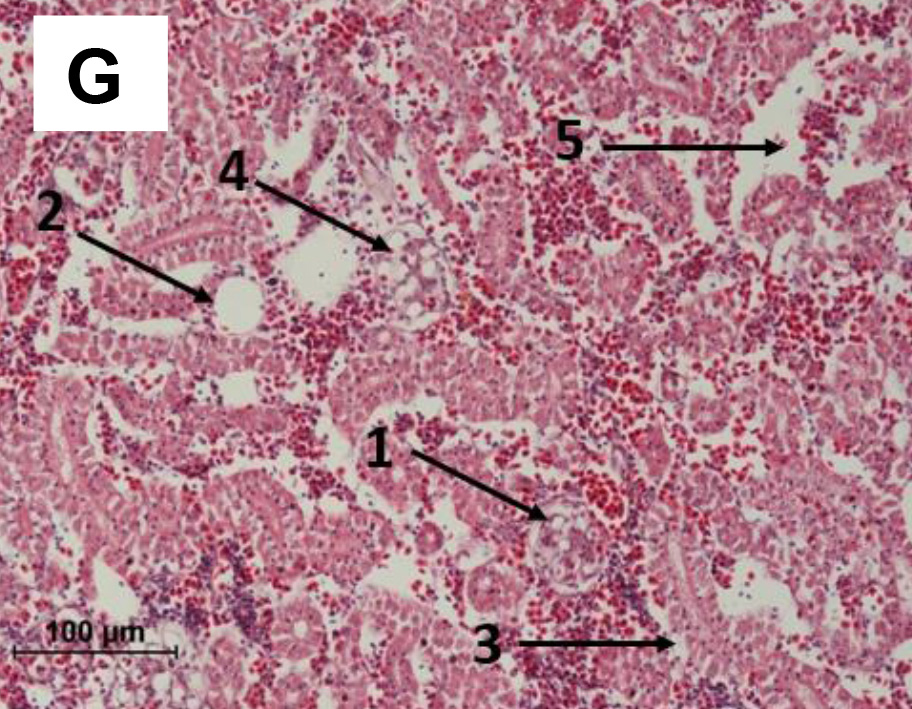
(A) Normal kidney of *C. gariepinus* (0 mg/L MCLE) with 1. Glomerulus, 2. Bowman’s space well defined, 3. Proximal tubule, 4. Distal tubule, 5 Bowman’s capsule, 6. Hematopoietic tissue; (B) 60 mg/L MCLE with 1. Haemorrhage; (C) 80 mg/L MCLE with 1. Haemorrhage, 2. Tubular degeneration; (D) 100 mg/L MCLE with 1. Shrinkage of glomerulus, 2. Vacuolation and hydropic degeneration; (E) 120 mg/L MCLE with 1. Nuclear alterations, 2. Vacuolation and hydropic degeneration; (F) 140 mg/L MCLE with 1. Haemorrhage, 2. Tubular necrosis; (G) 160 mg/L MCLE with 1. Severely necrotic kidney glomerulus, 2. Vacuolation, 3. Tubular degeneration, 4. Dilatation of glomerulus capillaries, 5. Hematopoietic tissue with red blood cells and poor tubular structural. H&E stain, 400× total magnifications.

No lesions were observed in non-treated fish liver (Fig. 4A). Meanwhile, alterations were observed in liver tissues at 60 mg/L MCLE, including congestion of sinusoids and vacuolation (Fig. 4B). A slight changes like plasma alteration, degeneration, and leukocyte infiltration in fish exposed to 80 mg/L MCLE (Fig. 4C). More alteration was found in higher MCLE concentration such as congested of central vein with poor hepatic cord structure (Fig. 4D) and hepatocytes focal necrosis (Fig. 4E), and even more severe in the higher MCLE concentration (Fig. 4F). For the kidney, a normal appearance was observed in the untreated kidney tissues (Fig. 5A). However, a slight change was observed with the increased plant concentration (Fig. 5B), including tubular congestion with red blood cells and hydropic degeneration (Fig. 5C), vacuolation, hydropic degeneration of tubular cells (Fig. 5D), and severe hydropic degeneration of tubular cells, haemorrhage and tubular necrosis (Figs. 5E and 5F).

## DISCUSSION

The histopathological evaluation of tissue and cells caused by toxicants is crucial to determining the health condition of organisms. In this present study, MCLE was exposed for 96 h to African catfish at sub-lethal doses, and the histological alterations in the gills, liver, and kidney were observed. In the GC-MS analysis, 37 compounds were detected in MCLE, with terpenes and flavonoids being the major compounds. Terpenes such as caryophyllene oxide (2.07%), 1H-imidazole, 4,5-diphenyl-18.60%, 2-isopropyl-10-methylphenanthrene (13.50%), and stigmasterol (1.02%) are among the higher peaks found in MCLE, which have also been reported previously (Noor *et al*. 2021; Białoń *et al*. 2019; Ukit *et al*. 2019), however, with a slight difference in quantity, which might be influenced by several geographical factors including method of extraction, harvesting period, part of the plant used and more (Kumari *et al*. 2014). These compounds may act synergistically or in a single compound to serve a variety of biological properties such as antibacterial (González *et al*. 2021; Moghrovyan *et al*. 2019), anti-inflammatory (Al-Khayri *et al*. 2022; Prado-Audelo *et al*. 2021), antioxidant (Ivanović *et al*. 2020; Amri & Hossain 2018), antifungal (Salomé-Abarca *et al*. 2020; de Lima *et al*. 2022), and many more.

An acute toxicity test of MCLE on African catfish has been conducted previously by Nagi *et al*. (2016), which resulted in a 96-hour LC_50_ of 127 mg/L. Following that, a sub-lethal toxicity test was performed in this current study to determine the histopathological effects on the gills, liver, and kidney of African catfish during the exposed time. Gills are a good indicator of water quality because of their large surface area and contacts with the external environment (Strzyzewska *et al*. 2016). The harmful chemical and biological substances in the water surroundings may influence the gills’ morphological changes and impairment of their functions. The results of this study revealed that histopathological changes such as epithelial lifting, oedema and hydropic degeneration were observed in fish gills exposed to lower concentrations of MCLE, but the effects increased proportionally with MCLE concentrations, such as lamellar disorganisation and necrosis. It can be speculated that these significant alterations may be attributed to the high content of phytochemical compounds in MCLE. Although mucous secretion aids in the prevention of toxicants passing to the gill epithelium, these consequences may disturb the gas exchange process and reduce respiration (Nero *et al*. 2006). Similar findings were reported by Pal *et al*. (2012) after exposure of chlorpyrifos to common carp, *Cyprinus carpio*. The common alterations observed in all concentrations were epithelial lifting and hydropic degeneration, which increased proportionally with toxicant concentrations. Similar alterations were found in *Sparus aurata* following acute and chronic exposure to erythromycin and oxytetracycline (Rodrigues *et al*. 2019). These alterations may be induced by severe oedema, leading to a partial or complete fusion of the gill filament (Yancheva *et al*. 2016).

The liver is a primary organ for detoxification of toxicants, storage of nutrients, metabolism, and synthesis of enzymes; it therefore becomes highly vulnerable to toxicants (Rodrigues *et al*. 2019). High exposure to MCLE resulted in focal necrosis, vacuolation, increase in sinusoidal spaces, and poor hepatic cord structure. Vacuolation can happen due to biochemical disturbances leading to slightly glycogen flocculent and single/multiple lipid accumulation in hepatocyte cytoplasms (Yancheva *et al*. 2016). Similar observations were found in fish exposed to toxicants (Nunes *et al*. 2015; Rodrigues *et al*. 2017). Liver necrosis is primarily induced by oxidative stress generated by toxicants (Verma *et al*. 2007) and was only found in higher exposures of MCLE. It may lead to irreversible liver damage like pyknotic nucleus with a long period of exposure due to nucleus hyperactivity (Pal *et al*. 2012).

A significant pathological index in the kidney was mainly attributed to degenerative alterations such as tubular degeneration coupled with nuclear alterations, which indicates that the exposure of MCLE at 100 mg/L to 160 mg/L was toxic to fish even at a shorter time. Tubule degeneration and necrosis were also reported by Correia *et al*. (2020) after acute effects of cerium dioxide nanoparticles on *Oncorhynchus mykiss*. In addition, a significant circulatory disturbance was observed in the kidney, including haemorrhage and shrinkage of the glomerulus, suggesting that MCLE affects cell permeability and causes disturbance to fluid transport in and out of cells (Hadi & Alwan 2012). Finding was contradicted with Shivappa *et al*. (2015) where they discovered no severe changes was observed in liver and heart tissues of goldfish and clownfish after exposure to *M. cajuputi* extract product, *Melafix*.

## CONCLUSION

During short-term application, the exposure of MCLE at acute levels showed several mild and moderate histological alterations in gills, liver, and kidney of African catfish, possibly due to effect of phytochemical compounds contain in extract. Additionally, the effects were dose-dependent, and using high MCLE concentrations, especially above the LC_50_ value (i.e., 120 mg/L to 160 mg/L), severely damages the organs’ tissues and structural integrity, potentially leading to organ malfunction. To ascertain their effectiveness, more research in other aquatic animal species may prove useful, as different animals may behave differently when exposed to plant extracts.

## Figures and Tables

**Table 1:** Phytochemical compounds in MCLE identified by GC-MS analysis.

No.	RT (min)	Name of the compound	Molecular formula	Molecular weight	Peak area (%)
1	5.598	1-(1-methylethyl)-4-methyl-3-Cyclohexen-1-ol,	C_10_H_18_O	154.2493	0.25
2	5.655	Benzene, 1-methyl-4-(1-methylethenyl)-	C_10_H_12_	132.2023	0.38
3	5.724	3-Cyclohexene-1-methanol,.alpha.,.alpha.,4-trimethyl-, (S)-	C_10_H_18_O	154.25	0.35
4	8.957	Naphthalene, 1,2,3,5,6,7,8,8a-octahydro-1,8a-dimethyl-7-(1-methylethenyl)	C_15_H_24_	204.3511	0.19
5	9.048	Naphthalene, 1,2,3,4,4a,5,6,8a-octahydro-4a,8-dimethyl-2-(1-methylethenyl)	C_15_H_24_	204.3511	0.26
6	9.2941	Naphthalene, 1,2,3,5,6,8a-hexahydro-4,7-dimethyl-1-(1-methylethyl)	C_15_H_24_	204.3511	0.22
7	9.569	Cyclohexanemethanol, 4-ethenyl-.alpha.,.alpha.,4-trimethyl-3-(1-methylethenyl)	C_15_H_26_O	222.3663	0.52
8	9.912	1H-Cycloprop[e]azulen-7-ol, decahydro-1,1,7-trimethyl-4-methylene-, [1ar-(1a.alpha.,4a.alpha.,7.beta.,7a.beta.,7b.alpha.)]-	C_15_H_24_O	220.3505	2.26
9	9.981	Caryophyllene oxide	C_15_H_24_O	220.35	2.07
10	10.084	Guaiol	C_15_H_26_O	222.37	3.32
11	10.227	7-Methyl-6,8-bis(methylthio)pyrrolo[1,2]pyrazine	C_10_H_12_N_2_S_2_	224.34	15.43
12	10.398	Cycloisolongifolene, 8,9-dehydro	C_15_H_22_	202.3352	1.66
13	10.438	2-Naphthalenemethanol, 1,2,3,4,4a,5,6,7-octahydro-.alpha.,.alpha.,4a,8-tetramethyl-, (2R-cis)	C_15_H_26_O	222.3663	1.96
14	10.496	6-Isopropenyl-4,8a-dimethyl-1,2,3,5,6,7,8,8a-octahydro-naphthalen-2-ol	C_15_H_24_O	220.3505	1.14
15	10.644	2-Naphthalenemethanol, decahydro-.alpha.,.alpha.,4a-trimethyl-8-methylene-, [2R-(2.alpha.,4a.alpha.,8a.beta.)]-	C_15_H_26_O	222.3663	7.25
16	10.776	1H-Cycloprop[e]azulen-4-ol, decahydro-1,1,4,7-tetramethyl-, [1ar-(1a.alpha.,4.beta.,4a.beta.,7.alpha.,7a.beta.,7b.alpha.)]-	C_15_H_26_O	222.3663	1.42
17	10.810	Humulen-(v1)	C_15_H_24_	204.3510	0.97
18	10.956	7R,8R-8-Hydroxy-4-isopropylidene-7-methylbicyclo[5.3.1]undec-1-ene	C_15_H_24_O		0.70
19	11.148	2-Acetyl-5-chloro-3-methylbenzo(b)thiophene	C_11_H_9_ClOS	224.7066	5.45
20	11.280	Bicyclo[4.3.0]nonane, 7-methylene-2,4,4-trimethyl-2-vinyl-	C_15_H_24_	204.3510	0.81
21	11.308	6-Isopropenyl-4,8a-dimethyl-1,2,3,5,6,7,8,8a-octahydro-naphthalen-2-ol	C_15_H_24_	204.3510	0.77
22	11.743	1-(3-Chloromethyl-2,4,6-trimethylphenyl)ethanone	C_12_H_15_ClO	210.6999	1.43
23	11.783	p-isopropylphenetole	C_11_H_16_O	164.2441	0.21
24	12.064	2’,3’,4’,5’,6’-pentafluoroacetophenone	C_8_H_3_F_5_O	210.1	0.43
25	12.149	2-naphthalenemethanol, 1,2,3,4,4a,5,6,8a-octahydro-.alpha.,.alpha.,4a,8-tetramethyl-, [2R-(2.alpha.,4a.alpha.,8a.beta.)]-	C_15_H_26_O	222.3663	0.57
26	12.727	4H-1-benzopyran-4-one, 5-hydroxy-7-methoxy-2-methyl-	C_11_H_10_O_4_	206.19	0.59
27	12.956	Hexadecanoic acid, methyl ester	C_17_H_34_O_2_	270.4507	0.29
28	13.299	Hexadecanoic acid	C_16_H_32_O_2_	256.4241	0.42
29	13.591	9-carboxaldehyde-10-methylanthracene	C_16_H_12_O	220.27	11.13
30	13.643	9H-fluoren-9-ol, 9-butyl-	C_17_H_18_O	238.3242	3.04
31	13.780	1H-imidazole, 4,5-diphenyl-	C_15_H_12_N_2_	220.2692	18.60
32	13.940	2-isopropyl-10-methylphenanthrene	C_18_H_18_	234.34	13.50
33	14.495	3,7,11,15-tetramethyl-2-hexadecen-1-ol			0.50
34	14.695	9,12,15-octadecatrien-1-ol	C_18_H_32_O	264.4461	0.42
35	17.677	1H-indole, 5-methyl-2-phenyl-	C_15_H_13_N	207.2704	022
36	17.763	5-methyl-2-phenylindolizine			0.27
37	26.088	Stigmasterol, 22,23-dihydro-	C_29_H_50_O	414.7067	1.02
